# Perspectives and Practice of HIV Disclosure to Children and Adolescents by Health-Care Providers and Caregivers in sub-Saharan Africa: A Systematic Review

**DOI:** 10.3389/fpubh.2016.00166

**Published:** 2016-08-12

**Authors:** Oluyemisi Aderomilehin, Angella Hanciles-Amu, Oluwatobi Ohiole Ozoya

**Affiliations:** ^1^Department of Community and Family Health, University of South Florida, Tampa, FL, USA; ^2^Department of Global Health, University of South Florida, Tampa, FL, USA; ^3^Emergency and Trauma Center, Tampa General Hospital, Tampa, FL, USA

**Keywords:** HIV disclosure, children and adolescents, health-care providers, caregivers, sub-Saharan Africa, ART adherence

## Abstract

**Background:**

Sub-Saharan Africa (SSA) has the highest prevalence of HIV globally, and this is due to persistent new HIV infections and decline in HIV/AIDS-related mortality from improved access to antiretroviral (ART) therapy. There is a limited body of work on perspectives of health-care providers (HCPs) concerning disclosing outcomes of HIV investigations to children and adolescents in SSA. Most studies are country-specific, indicating a need for a regional scope.

**Objective:**

To review the current literature on the perspectives of HCPs and caregivers of children and adolescents on age group-specific and culture-sensitive HIV disclosure practice.

**Methods:**

Electronic database search in PubMed, Google scholar, and the University of South Florida Library Discovery Tool (January 2006 up to February 2016). Further internet search was conducted using the journal author name estimator search engine and extracting bibliographies of relevant articles. Search terms included “disclosure*,” “HIV guidelines,” “sub-Saharan Africa,” “clinical staff,” “ART,” “antiretroviral adherence,” “people living with HIV,” “pediatric HIV,” “HIV,” “AIDS,” “health care provider,” (HCP), “caregiver,” “adolescent,” “primary care physicians,” “nurses,” and “patients.” Only studies related to HIV/AIDS disclosure, HCPs, and caregivers that clearly described perspectives and interactions during disclosure of HIV/AIDS sero-status to affected children and adolescents were included. Independent extraction of articles was conducted by reviewers using predefined criteria. Nineteen articles met inclusion criteria. Most studies were convenience samples consisting of combinations of children, adolescents, HCPs, and caregivers. Key findings were categorized into disclosure types, prevalence, facilitators, timing, process, persons best to disclose, disclosure setting, barriers, and outcomes of disclosure.

**Conclusion:**

Partial disclosure is appropriate for children in SSA up to early adolescence. Caregivers should be directly involved in disclosing to children but they require adequate disclosure support from HCPs. Full disclosure is suitable for adolescents. Adolescents prefer disclosure by HCPs and they favor peer-group support from committed peers and trained facilitators, to reduce stigma. HCPs need continuous training and adequate resources to disclose in a patient-centered manner.

## Introduction

The HIV pandemic is one of the most severe public health challenges facing the world to date. This pandemic has grave economic implications, especially in high prevalent regions like sub-Saharan Africa (SSA) (1, 2). The global HIV burden is estimated at 36.9 million cases; by the end of 2014, approximately 2 million new cases and 34 million deaths were attributed to AIDS-related causes (3–6). Recent data indicate that the top 10 ranking of HIV/AIDS cases by country is populated by countries in SSA (5, 6). In 2015, SSA contributed to 70% of new cases globally (5, 6). Factors contributing to the prevalence of HIV in SSA include improved access to antiretroviral (ART) medications and the resultant decline in mortality, while new infections from HIV/AIDS persist (1, 7).

Majority of new HIV infection cases occur in low- and middle-income countries that lack properly defined guidelines or resources to equip HCPs (3, 8, 9). HIV disclosure may be one of the critical links between new infections and the sustained high prevalence in SSA (10, 11). Inadequate health-care provider (HCP) training in HIV disclosure and testing services appear to contribute to new cases. Unfortunately, limited body of work exists on the prevalence and practice of disclosure by HCPs in SSA (7). Over the last 15 years, there has been a 35% decrease in global HIV infections and a 58% decrease among children, yet more than 54% of children currently infected may be unaware they have the disease (6, 12). A study on resource-limited countries that had available disclosure rates (Ghana, Kenya, and Ethiopia) reported rates that varied from 11 to 38% (11, 13, 14). This variability is consistent with studies in resource-rich countries where disclosure rates to children range widely from 10 to 77% (15–17). Furthermore, HIV disclosure practices in SSA remain complex due to the immense influence of culture, politics, and limited HIV surveillance (5, 10, 18). Disclosure rates in high prevalence regions need to be evaluated and improved drastically in a timely manner as HIV disclosure may be a key factor in reducing the risk of acquiring new infections, adherence to ARTs, and practice of safe sexual behaviors (7, 10, 19, 20).

Taken together, the incidence of HIV infection in SSA may be reduced by understanding the perspectives and roles of HCPs and caregivers in disclosing laboratory HIV test outcomes to children and adolescents in this region. In addition, studies in this region are country-specific; therefore, evaluating the perspectives of HCPs and caregivers across countries may provide more insight to achieve more reduction in new HIV infections. To determine the perspectives of HCPs and caregivers on age group-specific and culturally sensitive HIV disclosure practices in SSA, a systematic review of the perspectives and current patterns of HIV disclosure among HCPs and caregivers of children and adolescents was conducted.

## Methods

We searched for quantitative, qualitative (focus groups, interviews, and surveys), and mixed methods studies on HIV disclosure involving HCPs and caregivers in SSA from January 1, 2006 to February 28, 2016. English language restriction was imposed. Study participants included combinations of children and adolescents, HCPs and caregivers, or only caregivers or HCPs. Disclosure was categorized as full disclosure, partial disclosure, and non-disclosure (12, 21). Full disclosure is complete disclosure of HIV status with the term “HIV” appropriately used (11, 12, 21). In this case, the potential causes, transmission, and impact of the disease were discussed and treatment was clarified. Partial disclosure was performed similarly to full disclosure but the terms “HIV” or “AIDS” were excluded despite describing the morbidity and mortality from the disease (12, 21, 22). Other aspects present in full disclosure may also be excluded, for example, counseling (20, 21). Non-disclosure signifies no disclosure. In this case, individuals are not provided any information about their positive HIV status (12, 21).

The term “health-care providers” refers to health-care professionals, such as clinical staff, primary care physicians, nurses, midwives, and any health personnel, providing patient care in a clinical setting. A caregiver includes parents, family members, or individuals caring for a child or adolescent living with HIV/AIDS in a non-clinical/professional capacity. Articles were selected on application of the following inclusion criteria: social science work that examined HIV disclosure through qualitative or quantitative studies and mixed methods that clearly described perspectives and interactions among HCPs, caregivers, and infected children and adolescents.

### Search Strategy and Search Procedure

A computer-assisted systematic review was conducted, and extraction of articles was independently performed by the three authors who have expertise in community and family health, global health, epidemiology, and HIV management. Disagreements between reviewers were resolved by consensus. Three major electronic databases were searched using dates January 1, 2006 to February 28, 2016. These bibliographic databases included PubMed, Google Scholar, and the University of South Florida (USF) Library Discovery Tool. For USF Library Discovery Tool and PubMed, the term “HIV AIDS disclosure” was searched, then the Boolean operator and last set of terms “health-care provider” OR “caregiver” OR “patient” OR “adolescent” were added. Finally, the inclusion criteria were applied.

**Figure 1:**
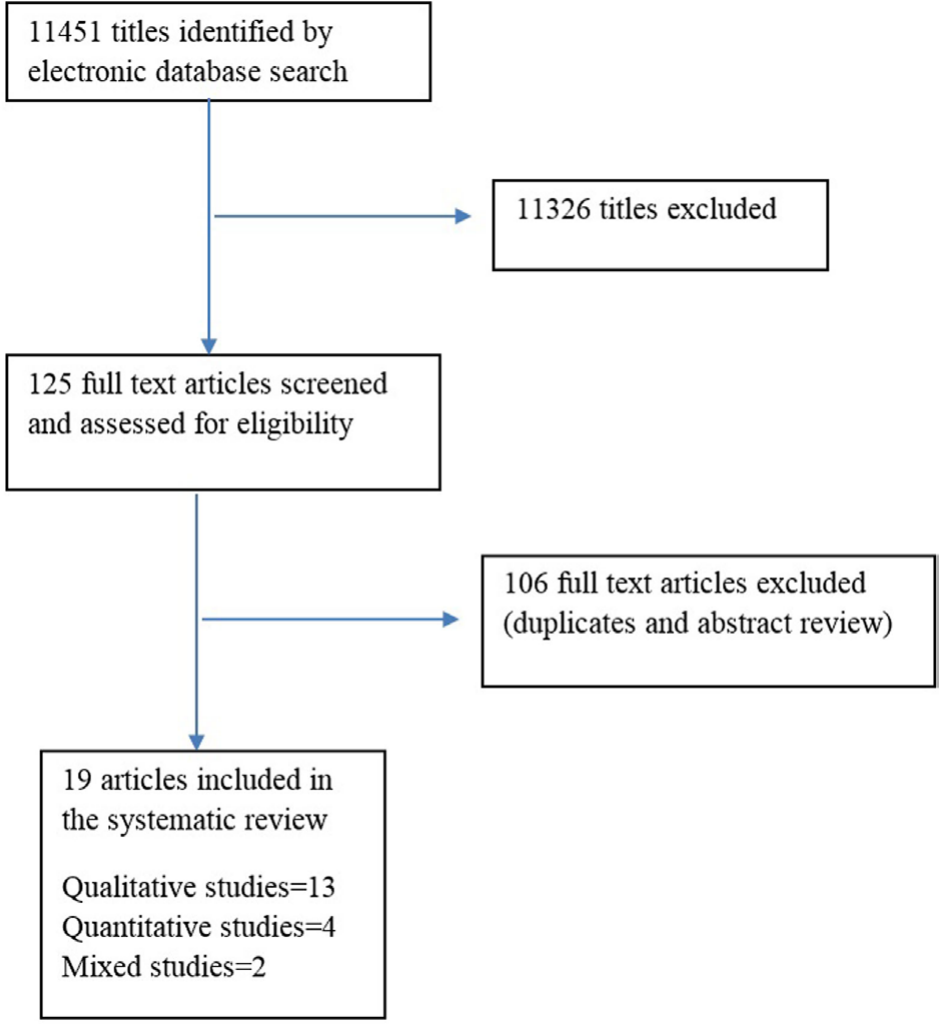
**Flow chart of HIV disclosure systematic review search process**.

For Google Scholar, the term “HIV disclosure” was searched then “AIDS disclosure” and “sub-Saharan Africa” were added. Next, the Boolean operator and keyword “health-care providers” OR “caregiver” OR “patient” OR “pediatric” OR “health-care professionals” were added. Finally, the inclusion criteria were applied. An additional internet search was conducted in Journal Author Name Estimator database (JANE) using the proposed search terms and their variants. Additional articles were cross referenced for further review of all the articles, and some articles were excluded either because they did not meet the inclusion criteria of the review or they were duplicates. Figure 1 shows a flow diagram of the search process.

Variants of keywords for HCP, caregiver, HIV and AIDS, and disclosure were also used. The search strategy was limited to articles that were accessible through the USF library. Full-text articles of all selected studies were retrieved, and if an article was selected, the bibliographic references were examined for additional relevant studies. Caregivers, HCP, and patients were combined with the use of the Boolean operator “OR.” The above searches, based on the two main interest HIV/AIDS disclosure and SSA, were combined using the Boolean operator “AND.”

### Data Extraction

A data extraction sheet was developed by two of the authors (Oluyemisi Aderomilehin and Angella Hanciles-Amu) and reviewed by the third author (Oluwatobi Ohiole Ozoya). A total of 19 articles met the inclusion criteria. First author, year and title of publication, country of study, population characteristics, study design, and theme topics were included in the sheet (Table 1). A summary of the results from included studies that were relevant to perspectives and practice of HIV disclosure to children and adolescents are detailed in Table 2.

**Table 1:** **Study characteristics**.

	Study	Title	Location	Population sample size	Study design and methods of data collection	Key findings
1	Mburu et al. (23)	Adolescent HIV disclosure in Zambia: barriers, facilitators, and outcomes	Zambia	223 164 adolescents 21 parents/caregivers 38 HCPs	Qualitative (interviews and focus group discussions)	Barriers Outcomes of HIV disclosure

2	De Baets et al. (21)	HIV disclosure and discussions about grief with Shona children: a comparison between health-care workers and community members in Eastern Zimbabwe	Zimbabwe	195 131 community members 64 HCPs in primary/rural health centers	Quantitative (anonymous survey)	Age of disclosure Best person to disclose to child

3	Demmer (24)	Experiences of families caring for an HIV-infected child in KwaZulu-Natal, South Africa: an exploratory study	South Africa	25 13 caregivers (mothers/females with a biological child sick from HIV/AIDS) 12 HCPs of children and families living with HIV/AIDS.	Qualitative (in-depth interview and semi-structured interview)	Barriers to disclosure

4	Gyamfi et al. (25)	Benefits of disclosure of HIV status to infected children and adolescents: perceptions of caregivers and health-care providers	Ghana	118 118 caregivers of HIV-infected children and adolescents. 10 key informants–HCPs and volunteer workers	Mixed method (quantitative and qualitative)	Best person to disclose Proportion Benefits of disclosing

5	Kajubi et al. (26)	Communication between HIV-infected children and their caregivers about HIV medicines: a cross-sectional study in Jinja district, Uganda	Uganda	394 394 children and their caregivers	Quantitative (cross-sectional survey)	Disclosure communication pattern Age (full/partial disclosure)

6	Kidia et al. (27)	HIV-status disclosure to perinatally-infected adolescents in Zimbabwe: a qualitative study of adolescent and health-care worker perspectives	Zimbabwe	46 31 perinatally infected adolescents 15 HCPs	Qualitative (in-depth interviews with adolescents; focus groups with HCPs)	Best person to disclose Disclosure setting Support

7	Midtbo et al. (28)	How disclosure and antiretroviral therapy help HIV-infected adolescents in sub-Saharan Africa cope with stigma	Botswana	14 12 adolescents 2 HCPs	Qualitative (interviews and observation)	Stigma Coping following disclosure
			Tanzania	19 16 adolescents 3 HCPs		

8	Moodley et al. (29)	Paediatric HIV disclosure in South Africa – caregivers’ perspectives on discussing HIV with infected children	South Africa	174 caregivers and children living with HIV	Qualitative (semi-structured interviews)	Disclosure proportion Age of child at disclosure Best person Facilitators Barriers

9	Kiwanuka et al. (30)	Caregiver perceptions and motivation for disclosing or concealing the diagnosis of HIV infection to children receiving HIV care in Mbarara, Uganda: a qualitative study	Uganda	40 Primary caregivers of HIV-infected children receiving HIV care but ignorant of their HIV status	Qualitative (in-depth interviews)	Disclosure-single event/process? Benefits and barriers

10	Lorenz et al. (31)	Caregivers’ attitudes towards HIV testing and disclosure of HIV status to at-risk children in rural Uganda	Uganda	28 Caregivers of HIV-positive children	Qualitative (semi-structured interviews)	Facilitators to testing child Age at disclosure Barriers Type of disclosure Support

11	Beima-Sofie et al. (32)	Using health provider insights to inform pediatric HIV disclosure: a qualitative study and practice framework from Kenya	Kenya	21 HCPs	Qualitative (interviews)	HCPs disclosure practices Family-centered disclosure Best Person to disclose Outcomes of disclosure

12	Mumburi et al. (33)	Factors associated with HIV-status disclosure to HIV-infected children receiving care at Kilimanjaro Christian Medical Centre in Moshi, Tanzania	Tanzania	236 211 parents or caregivers and their children 25 HCPs	Quantitative (cross-sectional with structured questionnaires)	Proportion of disclosure Age of disclosure Caregiver support

13	Myer et al. (34)	Health-care providers’ perspectives on discussing HIV status with infected children	South Africa	40 Health-care providers at a large pediatric clinic	Qualitative (semi-structured interviews)	Transition from partial disclosure To full disclosure in children Best person to disclose

14	Corneli et al. (35)	The role of disclosure in relation to assent to participate in HIV-related research among HIV-infected youth: a formative study	Democratic Republic of Congo	72 19 adolescents living with HIV 36 parents and caregivers 17 HCPs	Qualitative (semi-structured interviews)	Age of disclosure to children

15	Gachanja and Burkholder (36)	Model for HIV disclosure of a parent’s and/or a child’s illness	Kenya	34 12 children (7 HIV+ and 5 HIV− between 8 and 17 years) 16 HIV+ parents/caregivers 6 HCPs	Qualitative	Facilitators/motivation to disclose Age of disclosure Associated emotions Benefits Negative outcomes

16	Odiachi and Abegunde (37)	Prevalence and predictors of pediatric disclosure among HIV-infected Nigerian children on treatment	Nigeria	110Parents/caregivers of HIV-infected children	Quantitative (semi-structured interview)	Prevalence Age of disclosure Facilitators Barriers

17	Toska et al. (38)	Sex and secrecy: how HIV-status disclosure affects safe sex among HIV-positive adolescents	South Africa	858 Adolescents and their caregivers	Mixed methods [qualitative (interviews, focus group discussions and observations with 43 HIV-positive teenagers and their HCPs; quantitative interviewed using standardized questionnaires)]	Prevalence of disclosure Benefits Barriers

18	Vaz et al. (39)	Patterns of disclosure of HIV status to infected children in a sub-Saharan African setting	DR Congo	201 Primary caregivers of children 5–17 years old in an HIV pediatric care and treatment program	Qualitative (structured interviews)	Proportion of disclosure Facilitators Benefits Caregiver support in disclosure

19	Watermeyer (40)	‘Are we allowed to disclose?’: a health-care team’s experiences of talking with children and adolescents about their HIV status	South Africa	23 HCPs	Qualitative (focus groups)	Barriers to disclosure HCPs disclosure practices

**Table 2:** **High level summary of selected articles**.

Mburu et al. (23) **Barriers and outcomes of HIV disclosure** Barriers – local norms that deter parents from communicating with their children about sexuality; fear of HIV stigma; and an underlying presumption that adolescents would not understand the consequences of a HIV diagnosis on their lives and relationships Outcomes: individual level – anxiety, depression, and self-blame after disclosure. Interpersonal level – disclosure created opportunities for adolescents to access adherence support and other forms of psychosocial support from family members and peers. At the same time, it occasionally strained adolescents’ sexual relationships, although it did not always lead to rejection	Watermeyer (40) **Barriers to disclosure and HCPs disclosure practices** Barriers – complexity of the disclosure process, confusion, hesitancy, and ethical dilemmas regarding disclosure practices Disclosure practices among HCPs – tensions were noted within the team which seem linked to professional hierarchies. Counselors and nurses preferred an indirect approach of encouraging caregivers to disclose to their children and providing psychosocial support, while doctors tended to become more directly involved in disclosing to children out of a sense of duty, legal responsibilities, and knowledge of the child’s rights	Demmer (24) **Barriers to disclosure** Stigma to HIV/AIDS in South African society made disclosing the child’s HIV status very difficult. There was concern about the reaction of partners and family members. Women were afraid of being blamed and abandoned. Stigma resulted in delayed testing of child and delayed treatment. Conspiracy of silence surrounding the child’s HIV status prevailed in many households. Teachers and principals were usually not informed about the child’s HIV status for fear of discrimination Perceived immature cognitive development of child, non-disclosure to most of the children who were under 10 years of age Caregiver anxiety over future disclosure to their child

De Baets et al. (21) **Age of disclosure and Best person to disclose to child** Age of disclosure – partial disclosure from the age of 10.8 (±4.2) years and full disclosure from the age of 14.4 (±4.5) years. HCPs openness to disclosure – compared to community members, health-care workers were significantly more open to full disclosure and disclosure at a younger age, but were slightly less open to discussing grief Best/preferred person to disclose – HCP in 56% of the responses or family member in up to 52%. The most commonly preferred family members – father’s sister (up to 37%) and grandmother (up to 40%) rather than the partner (up to 15%). Southern African family dynamics may hinder a mother initiating HIV disclosure and discussions about grief, even though she is traditionally present during HIV diagnosis, counseling, and health education. A more culturally adapted approach than the standard western “couple approach” may thus be required	Gyamfi et al. (25) **Best person to disclose, proportion, benefits of disclosing and elements needed in caregiver support** Most appropriate person to disclose to infected child – caregiver (47.5%) with the help of the HCP, caregiver alone (34.7%), and HCP (17.8%) Proportion of disclosure – to infected children and adolescents (48.8%), to mother of child (25.6%), and other family members (25.6%) Benefits of disclosure: yes (89%), no (11%). Most (46.6%) – improved adherence to medication and 31.4% – reported it promoted healthy and responsible sexual behavior when the child became an adolescent; 16.9% – made the children and adolescents more responsive to their health needs; and 5.1% – helped improve the mental and psychological health of the caregiver and/or the child Support elements: the main supports required by caregivers during disclosure included biomedical information, emotional and psychological support, and practical guidelines regarding disclosure	Kajubi et al. (26) **Disclosure communication pattern and age (full/partial disclosure)** 79.6% of the caregivers reported that they explained to the children about the medicines, but only half (50.8%) of the children were aware the medicines were for HIV. Older children aged 15–17 years were less likely to communicate with a caregiver about the HIV medicines in the preceding month (OR 0.5, 95% CI 0.3–0.7, *p* = 0.002). Children aged 11–14 years (OR 6.1, 95% CI 2.8–13.7, *p* < 0.001) and 15–17 years (OR 12.6, 95% CI 4.6–34.3, *p* < 0.001) were more likely to know they were taking medicines for HIV compared to the younger ones The least common reported topic of discussion between children and caregivers was “what the medicines are for” while “the time to take medicines” was by far the most mentioned by children

Kidia et al. (27) **Best person to disclose, disclosure setting and support** Health-care workers encouraged caregivers to initiate disclosure in the home environment In contrast, many adolescents preferred disclosure to take place in the presence of health-care workers at the clinic because it gave them access to accurate information as well as an environment that made test results seem more credible Adolescents learned more specific information about living with an HIV-positive status and the meaning of that status from shared experiences among peers at the clinic	Midtbo et al. (28) **Stigma and coping following disclosure** HIV-status disclosure enabled adolescents to engage effectively with their ART treatment and support groups, which in turn provided them with a sense of confidence and control over their lives Although the adolescents in both studies were still experiencing stigma from peers and community members, most did not internalize these experiences in a negative way, but retained hope for the future and felt pity for those untested and uninformed of their own HIV status	Corneli et al. (35) **Age of disclosure to children** Parents and caregivers favor disclosure to older children and adolescents than younger for HIV-related research HCPs and caregivers support disclosure to minors because it would improve adherence to treatment

Kiwanuka et al. (30)**Disclosure as a single event or process, benefits and barriers** Majority perceived disclosure as a single event rather than a process of gradual delivery of information about the child’s illness Benefits – potentially beneficial both to children and themselves, an opportunity to explain the parents’ role in the transmission of HIV to the children Barriers – caregivers desired to personally conduct the disclosure but most reported being over-whelmed with fear of negative outcomes (lack of self-efficacy in managing the disclosure process). Most cope by deception to avoid or delay disclosure until they perceive their own readiness to disclose	Lorenz et al. (31) **Facilitators to testing child, age at disclosure, barriers, type of disclosure and support** Facilitators – majority (96%) of respondents, the decision to test the child for HIV was due to existing illness in either the child or a relative Age at disclosure – most (65%) children were informed of their HIV status between the ages of 5 and 9, with the mean age of disclosure occurring at the age of 7 Barriers – existing stigma within community, doubts about cognitive understanding of child Full disclosure – general provision of HIV information typically began at the same age as disclosure Support in disclosing – two-thirds (64%) of the caregivers sought advice from an HIV counselor prior to disclosure	Beima-Sofie et al. (32) **HCPs disclosure practices, family-centered disclosure, best Person to disclose and outcomes** HCPs disclosure practices – providers had limited training but extensive experience in disclosure, endorsed individualized disclosure practices, invested substantial time on disclosure despite clinical burden Family-centered disclosure – child-centered disclosure but should respect caregiver fears and values Best person to disclose – caregiver support was provided to enable caregivers to be the person who ultimately disclosed HIV status to children Outcomes – unplanned or abrupt disclosure to children was reported to have severe and persistent adverse impact and was a stimulus to accelerate disclosure in scenarios when providers believed children may be suspecting their diagnosis

Moodley et al. (29) **Disclosure proportion, age of child at disclosure, best person, facilitators, and barriers** Only 9% had discussed HIV with the infected child Mean age of children who had been told their HIV status – 8.1 years Among the 73% of HIV-infected caregivers who had discussed their own infection with the child were more than 7 times more likely to have disclosed the child’s status to him/her (*p* = 0.07 after adjusting for age of the child). Age of disclosure – 12 years was the best age to tell a child about his/her HIV infection Best person to disclose to child – parent or primary caregiver (83%), 16% felt it would be best for a health-care provider (doctor, nurse or counselor). 25% reported they had discussed disclosure of the child’s HIV status with a health-care provider. Having discussed disclosure with a health-care provider was associated with disclosure to the child (*p* = 0.07). 96% of those who had not discussed disclosure with a health-care provider stated they would like to do Facilitators to disclosure – 98% of caregivers said they felt that the child has a right to know his/her HIV status, 90% gave reasons related to the child’s mental health. 70% of caregivers said that the availability of ART could make it necessary to discuss the child’s HIV status with him/her Barriers to disclosure – most caregivers (73%) said that they were afraid of the child discussing his/her HIV infection with other people	Gachanja and Burkholder (36) **Facilitators, age of disclosure, associated emotions, benefits, and negative outcomes** Motivation – chronic illness or acute illness presenting with AIDS-associated symptoms, desire to know HIV status, routine antenatal clinic attendance, or during general clinic visits where HCPs counseled HIV-positive parents that their children would eventually need to receive full disclosure of their own and/or their parents’ HIV statuses Age of disclosure – partial disclosure between 5–9 years of age; non-disclosure at <5 years Disclosure process and associated emotions – suspicion by non-diagnosed children of affected family members, guilt, and depression after disclosure Benefits – for caregivers, there was improved psychological health, increased support from their children, increased ability to take medications and attend clinic visits openly, improved medication adherence, and increased bonding with their children. For HIV-positive children, the benefits included increased independence; improved self-care, self-medication, and medication adherence; and a greater understanding about their HIV statuses, medications, and clinic attendance Negative outcomes – increased stress from rejection by family members, disrupted relationships. For HIV-positive children, there was drop in school performance; for HIV-negative children there was loss of normalcy in daily living, added responsibility at home	Odiachi and Abegunde (37) **Prevalence, age of disclosure, facilitators. and barriers** Prevalence – based on parents/caregivers’ accounts, 34 (30.9%) children knew that they were living with HIV, while 74 (67.3%) did not know Age at disclosure – mean age at disclosure was 10.47 years (± 2.62), with a median (range) of 10.00 (6–17) years Disclosure setting – 79.4% were disclosed at home by their parents/caregivers, rest at the hospital (5 by HCP; 2 accidental disclosure) Facilitators to disclosure – the most common reasons for disclosure were related to adherence issues – either to help prepare the children to take their medicines or that the child had refused to take his/her medicines (39.4%). This was followed by the child asking a lot of questions related to his/her health, frequent visits to the hospital, or why s/he was taking a lot of medicines even though s/he did not feel ill (27.3%) Barriers – most parents/caregivers did not disclose because the child was considered too young (84.0%) or will not be able to keep their HIV status a secret (10.7%). Multivariate logistic regression showed that only child’s age was a statistically significant predictor of status disclosure (OR 1.69, *p* = 0.002; 95% CI 1.21–2.34). There was no association between disclosure and self-reported adherence (*p* = 0.615).

Toska et al. (38) **Prevalence of disclosure, benefits, and barriers** Prevalence – 68.1% of the sample knew their status, 41.5% of those who were sexually active and in relationships knew their partner’s status, and 35.5% had disclosed to their partners Benefits – for adolescents, knowing one’s status was associated with safer sex (OR = 4.355, CI 1.085–17.474, *p* = 0.038). Neither knowing their partner’s status, nor disclosing one’s HIV-status to a partner, were associated with safer sex Barriers – HIV-positive adolescents feared rejection, stigma, and public exposure if disclosing to sexual and romantic partners. Counseling by health-care workers for HIV-positive adolescents focused on benefits of disclosure, but did not address the fears and risks associated with disclosure	Vaz et al. (39) **Proportion of disclosure, facilitators, benefits, and caregiver support in disclosure** Proportion of disclosure – about 50% of caregivers provided no information to their child about their health; 15% had given partial information without mentioning HIV, and 33% provided information that deflected attention from HIV, whether deliberately so or otherwise Facilitators and benefits – almost all caregivers said that the child should be told their status someday, and three-fourths reported having ever thought about what might lead them to tell. However, nearly one-third of caregivers saw no benefits to informing the child of her/his HIV status Caregiver support in disclosure – a majority of caregivers felt that they themselves were the best to eventually disclose to the child, but some wanted support from health-care providers	Mumburi et al. (33) **Proportion of disclosure, age of disclosure, and caregiver support** Proportion of disclosure – 53.1% non-disclosed, 24.6% partial disclosure, 22.3% knew their HIV status The mean age of disclosure was 10.6 years. Most of disclosed children were aged above 10 years (*p* < 0.001) and self-supervised in taking medication (OR 33.1; 95% CI 13.9–78.9). Children who got other support were more likely to be disclosed than those who did not (OR 2.4; 95% CI 1.1–5.0) Caregiver support – parents/caregivers who discussed with health-care providers about disclosure did disclose HIV status more often to their children (OR 4.4; 95% CI 2.2–8.7)

Myer et al. (34) **Partial disclosure to children and transition to full disclosure, best person to disclose** Disclosure to children – most providers felt that the optimal age for general discussions about an HIV-infected child’s health should happen around age 6, but that specific discussions regarding HIV infection should be delayed to a median of 10 years Best person to disclose – though most providers said that primary caregivers were the most appropriate individuals to lead disclosure discussions, there were strong views that caregivers require support from health-care providers		

## Results

All studies in the final selection included various types of studies ranging from 13 qualitative study design, including interviews and focus groups (23, 24, 27, 28, 30–32, 34–36, 39, 40), to 4 quantitative study design (21, 26, 33, 37) and 2 mixed methods design (25, 38). The 19 articles included studies conducted in 10 SSA countries. The sample sizes were variable, ranging from 21 to 858 (median 79). Most studies utilized small samples, with the largest four studies including 40 HCPs (34), 394 caregivers (26), and 858 children and adolescents (38). Three studies included only HCPs (32, 34, 40), and five studies included only caregivers (29–31, 37, 39). One study included both HCPs and community members with focus on HIV disclosure and discussions about grief (21). The three studies that included only HCPs-provided information on types of disclosure in practice and the most suitable person and setting to disclose (32, 34, 40). Some of the studies included caregivers living with HIV and others without HIV. Caregivers were involved in the care of HIV-positive children, adolescents, or family members (29–31, 37, 39). Estimation of disclosure prevalence was limited to two articles (37, 38). The studies explored perspectives on disclosure types, prevalence, facilitators, timing, process, persons best to disclose, disclosure setting, barriers, and outcomes of disclosure. This is described below.

### Types and Prevalence of HIV Disclosure

Disclosure was defined by mention of “HIV or AIDS” when explaining the illness to infected children and adolescents, implying full disclosure, otherwise, partial disclosure is the case (21, 24, 26, 33, 34, 38, 40). Non-disclosure was present in two studies where the children were neither aware of the illness nor the reason for taking medications (33, 39).

Only two studies reported on prevalence of HIV disclosure estimated at 30.9% among infected children and 68.1% among infected adolescents (37, 38). In these studies, the infected children and adolescents were aware of their HIV infection, indicating full disclosure. Three other qualitative studies reported less than half of caregivers who had disclosed to their children (25, 29, 39). Two studies reported non-disclosure by 50% of caregivers and simultaneously reported partial disclosure by 15–24.6% of caregivers (33, 39).

### Facilitators and Timing of Disclosure Type

Major facilitators for caregivers in initiating disclosure was knowledge of availability of ART therapy (29), view of disclosure as the right of the child and adolescent (29, 41) when adhering to ART therapy was a potential or actual problem for the infected child or there was frequent visits to health facilities despite absence of overt illness (37, 39). Other facilitators to HIV disclosure include persistent inquiries by the HIV-positive child or adolescent (36, 37), presence of chronic illness in the child or a family member (31, 39), or discovery at routine antenatal clinic attendance by infected mothers (36). A common predictor of the timing of disclosure to children was the age of the child; the age for disclosure varied widely among the studies and ranged from 5 to 15 years (21, 26, 29, 31, 33–37). Studies that reported a specific age of disclosure to children included 5 years (36), 7 years (31), above 10 years (33, 34, 37), 12 years (29), and 15 years (26, 35). Three studies distinguished age at full disclosure as above 10 years (34, 36), 14 years (21), and 15 years (26). Two studies specifically reported partial disclosure by caregivers at ages <10 years (21, 34, 36) and 15 years (26, 35).

### Process of HIV Disclosure

Irrespective of whether it was a HCP or caregiver being interviewed, some of the studies indicated that the process of disclosure can be complex (23, 24, 26, 29, 37–40). One study reported caregivers who perceived disclosure as a single event (30). Only one study reported different phases of disclosure that included pre-disclosure, disclosure, and post-disclosure phases of disclosure to children (36). Here, the parents of infected children were counseled to anticipate and resolve questions the children may have prior, during, and following disclosure (36). Some other studies reported caregivers who preferred to receive counsel from HCPs or HIV counselors before disclosing (29, 31, 39). From the time of diagnosis, some participants adopted partial disclosure till the children were perceived as ready to receive full disclosure (21, 34, 36). Only one of the studies clearly described the post-disclosure phase (36). In the post-disclosure phase, some of the children in the study had questions about transmission of the disease, HIV management, and eventual cure (36).

### Persons Best to Disclose

Persons best positioned to disclose varied in different studies and by the key participants asked. Majority of the studies that explored “persons best to disclose” favored disclosure by caregivers with support from HCPs (27, 29, 31–34). One study reported preference for HCPs disclosing rather than caregivers (25). Two studies among caregivers in South Africa and Uganda reported high preference for the parent or caregiver to disclose rather than HCPs (29, 30). A study in Uganda and Kenya indicated that HCPs support caregivers when they initiate disclosure (27, 32), but adolescents preferred HCPs to disclose rather than their caregivers because they had an opportunity for more accurate information about their disease (27). A study in South Africa indicated that caregivers preferred that the HCP or family members, especially the paternal aunt and grandmother disclose rather than the mother (21). Although some caregivers preferred to disclose to infected children themselves, they expressed fears of competence in disclosure; acceptance by the child; stigma from accidental disclosure by the child to others; and need for support (29, 31, 32, 34, 39).

### Approach to Disclosure

Perspectives of different groups of HCPs differed on approach to disclosure. Nurses and counselors shared preference for encouraging caregivers to disclose to their children and adolescents with adequate support (40), unlike doctors who preferred direct participation in disclosure from a professional and legal obligation (32, 40). HCPs experienced conflict between human rights or public health safety during disclosure and also required training in decision-making and to reduce inadvertent disclosure (37). When HCPs were to discuss grief or HIV diagnosis with children and adolescents, HCPs preferred full disclosure of HIV diagnosis to children at a younger age (6–15 years) rather than discussing grief. In contrast, caregivers preferred that children were older (10–19 years) since they were more open to discussing grief than HIV.

### Disclosure Setting

Disclosure setting was an important consideration in the disclosure process according to some of the studies. The health facility setting was preferred by HCPs because this provided an avenue for caregiver support during the disclosure process (32), and also gave adolescents the opportunity to obtain reliable answers to questions and interact with peers at the health facility (27, 28). For caregivers who preferred their HCPs to be involved in the disclosure process, the health facility was the natural setting where this occurred (29, 33, 34, 40). Where caregivers preferred to handle disclosure to their minors, the home setting was the natural environment (30).

### Barriers and Outcomes of HIV Disclosure

Barriers to disclosure by HCPs or caregivers included the fear of HIV stigma, uncertainty about cognitive development of children, and local traditions that limit discussion of sexuality (23, 24, 29, 36). Some studies described benefits from the disclosure process, and this included improved ART adherence and mental health of affected individuals (25, 35), opportunity to cope with the illness, and the associated stigma (27, 28, 30). By contrast, a study reported adolescents who experienced peer-stigma, anxiety, depression, and self-blame after knowing their positive status (28). Furthermore, some studies indicated that disclosure, especially premature or inadvertent disclosure, resulted in harmful outcomes such as stigma, discrimination, and abuse from family and community members (23, 28, 32, 36). Some caregivers coped by adopting a conspiracy of silence (24). Besides, a study in Democratic Republic of Congo reported up to one-third of caregivers who did not see any benefit in disclosing to children (39). In addition, a study involving HCPs and adolescents indicated that adolescents did not see any benefit in knowing their partner’s status nor disclosing to a partner (38). Rather, benefit was reported from knowing self-status as this informed safer sexual lifestyle (38). While one study suggested that disclosure may empower the child or adolescent to engage in safe sexual behaviors (25), another study indicated there was no correlation with sexual lifestyle but with medication adherence (38). Caregivers or other family members also had to disclose their HIV status to facilitate the communication of perinatal infection and ART adherence to infected children and adolescents (24, 29, 36). However, some caregivers expressed concerns that this process could generate both positive and negative outcomes (24, 29, 36).

## Discussion

The objective of this review was to explore the perspectives and practices of HCPs and caregivers in HIV disclosure to children and adolescents. Findings are categorized as follows: types and prevalence of disclosure; facilitators, timing and barriers; process of disclosure; persons best to disclose, approach to disclosure by HCP versus caregivers; suitable disclosure setting and outcomes of disclosure.

This review showed that the prevalence of full disclosure is relatively low for children compared to adolescents, which is higher (37, 38). However, the prevalence of HIV disclosure needs to be improved for both age groups because more disclosure will reduce the number of infected children and adolescents at risk of morbidity and mortality from poor adherence to ARTs (23, 28, 32, 36). Despite natural concerns of adults about a child’s ability to cope with disclosure, empirical studies show that children may be more resilient than assumed (42, 43), appropriate disclosure may enhance psychological stability of the child and improve adherence to ART therapy (28, 42, 44). For adolescents, more disclosure by HCPs or caregivers to infected adolescents directly will encourage safer sexual behaviors that reduce new HIV transmission rather than emphasizing adolescents disclosing to their partners (38).

In SSA, variation exists in the types of disclosure in practice, and this review has highlighted studies where participants utilized partial disclosure before full disclosure of HIV-positive status or adopted outright non-disclosure (21, 26, 33, 34, 36, 41). Most studies identified the age of a child as a key factor that determined the timing of disclosure. It is important to identify cues to disclosure to children early since appropriate training for HCPs and caregivers may enhance their readiness to disclose. Age, as a key factor, also emerged in a systematic review by Vreeman et al. on pediatric disclosure practices in resource-limited settings (20). Most of the studies in this review suggest that, in SSA, partial disclosure may be appropriate for children up to early adolescence (21, 22, 34, 36), and this is consistent with the report from WHO Guideline on HIV Disclosure Counselling for Children up to 12 years of Age (2011) (12, 43). Despite age as a key factor in disclosure to children, we recommend that consideration needs to be given to communication patterns within families, orphan status, cultural norms, influence of other family members living with HIV/AIDS, and involvement of the child in administering ART medications (33, 36, 37).

Again, this review indicated that the concept of HIV disclosure process is relatively understudied since few studies evaluated perception of HCPs and caregivers. Caregivers described the disclosure process as complex, and one study reported caregivers who viewed disclosure as a solitary event. To reduce the complexity associated with disclosure, it is important to disseminate HIV disclosure as a process originating from the time of initial diagnosis to events beyond disclosure. Here, a series of dialog is made with the child about the terms, course of disease, relationship with others, self-care, and medications over time (36, 45). The dialog between caregivers and their children may be strengthened by providing focused counseling to caregivers throughout the disclosure process (42, 43, 46). Scientific research has demonstrated that the process-oriented approach results in less strain on caregivers and better outcomes for infected children and adolescents (28, 36, 42, 44, 47).

From the selected studies, there was evidence that both HCPs and caregivers perceived the most suitable individuals to disclose to children in SSA are caregivers, with support from HCPs throughout the process (27, 31, 32, 35, 37, 41). The WHO guideline on disclosure counseling to children under 12 years of age (2011) indicated that there is no evidence for either HCPs or caregivers disclosure as the best to disclose, but emphasized that disclosure should be in the best interest of the child (12). Adolescents had a preference for disclosure by the HCP because they received more biomedical information and could cope better with the disease than information received from their caregivers alone (27). Adolescents also indicated that peer-group support was important to them (23, 27). This suggests that the support needs of adolescents during the disclosure process differ from those of younger children in the region.

The health-care setting also plays a critical role in disclosure practice. Among HCPs, physicians tended to disclose more directly from a legal, moral, and ethical obligations compared to other workers who emphasized provision of support to caregivers who should disclose directly to infected children (40). As more HCPs disclose, it is critical to expand the locations where their services can be accessed by affected families. A review by Obermeyer and colleagues provided evidence that increased HIV/AIDS services in areas with limited services may facilitate disclosure and reduce stigma in the affected communities (46). Creating more health-care centers for HIV/AIDS services is an additional cost in resource-limited parts of the SSA, but the World Health Organization, in 2015, recommended HIV counseling and testing by trained lay providers (community health workers) to reduce the cost of providing needed services and to increase access to care (48). Some success is being recorded with lay providers in this regard, but this strategy requires more studies and careful review of existing policies to seamlessly integrate lay providers in provision of counseling and testing services (49–51).

Stigma and discrimination remain a persistent threat to the potential benefits of HIV disclosure. Sadly, local norms that deter discussion of sexuality also indirectly limit HIV disclosure by their HCPs and caregivers to children and adolescents (23). While advocating for any type of disclosure, it is important to consider individual rights and safety carefully balanced with public health safety.

### Limitations

Some limitations are inherent in this review. For example, although our review focused on SSA, not all countries in this region had adequate research available on HIV disclosure by HCPs and caregivers. SSA was only represented by 10 countries out of 46 (Botswana, Democratic Republic Congo, Ghana, Kenya, Nigeria, South Africa, Tanzania, Uganda, Zambia, and Zimbabwe). Four studies performed prior to or during the period (2008) of increased access to ART in SSA may not have clearly depicted the current disclosure process and practices because of the recent discovery of a relationship between access to ART and HIV disclosure (7, 29, 34). Reported perspectives and practices may slightly differ in the health-care setting or living situation of participants; therefore, caution must be applied in interpretation of the findings and in making generalizations.

The quality of the studies varied especially with majority consisting of convenience sampling. Restricting the search to English-language publications may have excluded studies or participants from non-English speaking parts of SSA. Paucity of research on this topic limited the discussion of several other issues, such as specific disclosure communication and practices. Studies on prevalence and proportions of disclosed patients were largely for children and relatively less for adolescent, again limiting the scope of the review.

### Implication

Because timing is an important factor to consider, best practices would have to include training of HCPs and caregivers to identify appropriate timing for disclosure, especially at the early phase of diagnosis (43). Continuing education and training that incorporate health policies and are amenable to local norms may contribute to the efficacy of HCPs when addressing barriers to HIV disclosure in their communities (48). Since the advent of more available ART, the focus has been on prompt diagnosis, prevention of mother-to-child transmission (PMTCT), partner disclosure, and testing with some success in HIV risk behavioral changes. However, success in individual behavior change will remain a challenge where the route of transmission involves at least two individuals or children infected perinatally. Findings from this review indicate the importance of broadening the scope of current HIV intervention programs to include adequately informed and appropriate disclosure. Therefore, long-term HIV/AIDS program funding should provide for resources to ensure that disclosure does not stop at informing individuals (e.g., infected mothers) of their HIV status or encouraging ART adherence; rather these resources should also be channeled to ensure adequate counseling and support after disclosure to minimize stigma, abuse, poor ART adherence, and transmission of new HIV infection.

Peer groups are helpful to adolescents but require participation of motivated members living with HIV and ought to be facilitated by trained personnel (27, 28). Although two studies among adolescents referred to peer support as a coping method for this population, stigma from peers was a reality in other studies (27, 28, 33). Therefore, when peer groups are being facilitated by HCPs, caution must be exercised to ensure appropriate groups are created and proactive measures undertaken to minimize stigma from peers.

The role of HCPs in HIV disclosure is challenged by insufficient number of HCPs relative to the sub-Saharan population (52, 53). Poor training, difficult working conditions, and increasing emigration to urban areas and developed countries has contributed to HCP shortage in about 31 countries in SSA (52, 53). The shortage in HCPs who daily endure stressful working conditions inadvertently impinge on optimal disclosure practices. To reduce the burden on HCPs, trained lay providers (community health workers) should be integrated in the disclosure process. Also, the responsibility to disclose can be shared equally by all cadres of HCPs trained in HIV disclosure, and team work should be driven by a patient-centered approach to caring for infected children and adolescents. An extension of this collaboration is a family-centered approach, where the health-care team collaborates with family members (or caregivers) directly involved in the care of the child at home or with individuals selected by family members to represent them (21, 43).

The ethical issues involved in disclosure have contributed to the complexity associated with establishing specific HIV disclosure guidelines in SSA (7, 8). To get the best result from disclosure, HCPs need to disclose in the most ethical and culturally competent manner with full inclusion of patient confidentiality. Broad guidelines may be more effective for SSA as this lends itself to accommodate the cultural diversity of each country and even regions within specific countries. From these broad guidelines, hospital or clinic leaders can provide specific guidelines based on the local context as this would be helpful to HCPs and caregivers when taking care of their patients and children, respectively. Beyond the perspectives of HCPs and caregivers, the perspectives of people and communities with high HIV burden need to be incorporated in revising or creating local guidelines on HIV disclosure. Other factors, such as facilitators and barriers associated with HIV disclosure to children and adolescents, need to be factored into new guidelines. The degree to which these broad factors are considered in the development and implementation process will likely result in a more successful adoption of disclosure guidelines.

## Conclusion

This review evaluated the perspectives of HIV disclosure and practices currently used in delivering age- and culture-sensitive HIV disclosure to children and adolescents by health caregivers or caregivers in SSA. Further evidence has been generated on the emerging topic of HIV disclosure in this region following the advent of ART. Partial disclosure is appropriate for children in SSA up to early adolescence. Caregivers should be involved in disclosing to children, and they require adequate disclosure support from HCPs. On the other hand, full disclosure is suitable for adolescents. Adolescents prefer disclosure by HCPs and they favor peer-group support from committed peers and trained facilitators, to reduce stigma. HCPs need continuous training and adequate resources to disclose in a patient-centered manner. Community members need education in HIV stigma reduction.

The evidence-based information from this review informed the following recommendations. First, caregivers and HCPs require collaborative training to ensure the best interest of infected children and adolescents are addressed throughout the disclosure process. Second, public health education should promote HIV/AIDS as a shared burden and create opportunities for community members to accept families of children and adolescents living with HIV to reduce stigma in the communal life. Inclusion of trained lay providers selected from affected communities may also ensure more culturally acceptable management of HIV disclosure in these communities. Third, family counseling and community education that encourages discussion of sexuality within local norms will empower children and adolescents to make better informed sexual health- or HIV-related choices. Finally, more studies are needed to determine the role of social determinants in HIV disclosure practices in resource-limited communities.

## Author Contributions

All the authors made substantial contributions to the conception and design of the work and in the acquisition, analysis, and interpretation of data for the work. They also drafted the work or revised it critically for important intellectual content. Final version of the article to be published was approved by all the authors. They also agree to be accountable for all aspects of the work in ensuring that questions related to the accuracy or integrity of any part of the work are appropriately investigated and resolved.

## Conflict of Interest Statement

The authors declare that the research was conducted in the absence of any commercial or financial relationships that could be construed as a potential conflict of interest.
